# A protocol for a systematic review investigating the factors influencing the statistical planning, design, conduct, analysis and reporting of trials

**DOI:** 10.12688/hrbopenres.13068.2

**Published:** 2020-09-24

**Authors:** Marina Zaki, Marie Galligan, Lydia O'Sullivan, Declan Devane, Eilish McAuliffe

**Affiliations:** 1 University College Dublin, Centre for Interdisciplinary Research, Education, and Innovation in Health Systems (IRIS Centre), School of Nursing, Midwifery and Health Systems, University College Dublin, Dublin 4, Ireland; 2 Health Research Board – Trials Methodology Research Network (HRB-TMRN), School of Nursing and Midwifery,, National University of Ireland, Galway, Galway, Ireland; 3 School of Medicine and Medical Science, University College Dublin, Dublin 4, Ireland; 4 School of Nursing and Midwifery, National University of Ireland, Galway, Galway, Ireland; 5 Evidence Synthesis Ireland, National University of Ireland, Galway, Galway, Ireland

**Keywords:** Clinical trials, statistics, systematic review, protocol

## Abstract

Trials can be defined as prospective human research studies to test the effectiveness and safety of interventions, such as medications, surgeries, medical devices and other interventions for the management of patient care. Statistics is an important and powerful tool in trials. Inappropriately designed trials and/or inappropriate statistical analysis produce unreliable results, with limited clinical use. The aim of this systematic literature review is to identify, describe and synthesise factors contributing to or influencing the statistical planning, design, conduct, analysis and reporting of trials. This protocol will describe the methodological approach taken for the following: conducting a systematic and comprehensive search for relevant articles, applying eligibility criteria for the inclusion of such articles, extracting data and information, appraising the quality of the articles, and thematically synthesizing the data to illuminate the key factors influencing statistical aspects of trials.

## Abbreviations

FDA: Food and Drug Association; ICH GCP: International Conference on Harmonisation Good Clinical Practice; PRISMA: Preferred Reporting Items for Systematic Reviews and Meta-Analyses; WHO: World Health Organization

## Introduction

Clinical trials are research studies that test the safety and efficacy of novel treatments and interventions. Their effects on the health outcomes of humans are evaluated prospectively ( WHO, 2020). Such medical interventions include drugs, cells (and other biological products), surgical procedures, medical devices, behavioural treatments, radiological procedures and measures for preventative care ( WHO, 2020). Findings from trials have the potential to change clinical practice. For this reason and to minimise harm to patients, trials must be conducted to a very high standard.

The Merriam-Webster Dictionary describes statistics as " *a branch of mathematics dealing with the collection, analysis, interpretation, and presentation of masses of numerical data"* ( Merriam-Webster Dictionary, 2020). To put this definition into context, it implies: refining the study design in order to efficiently address the study’s research hypothesis while minimising bias, defining data to be collected, appropriately analysing data collected, and interpreting results in such a way as to facilitate clinical decision making.

Clinical data management (CDM) facilitates the statistical aspects in a trial. CDM activity in a trial involves the appropriate collection, management, access to and cleaning of clinical research data. The integrity of statistical analysis in a trial ultimately depends on the quality of the data that is available for analysis ( Adams-Huet & Ahn, 2009; Krishnankutty *et al.*, 2012; Nesbitt, 2004, p.135). The validity of a clinical research study is therefore not solely judged on its results, but also on how the study itself was designed and conducted ( Adams-Huet & Ahn, 2009). The key team members responsible for the quality of statistical outputs from a trial are statisticians (/biostatisticians), clinical data managers and principal investigators (PIs). It is important to note the importance of, not only their roles and responsibilities, but their qualifications, experience, and training and how they work with other team members, and the potential impact these may have on the statistics of a trial.

There is also a relationship between statistics and ethics in trials. Ethical issues can affect not only the design of a trial, but all stages. If the statistical considerations of a trial are inadequate, the research will be unethical. This misuse of statistics in the clinical research field may have consequences for trial participants ( Altman, 1980), and other resources, including researchers’ time and effort. It is also unethical to publish and disseminate statistical results that may be misleading ( Altman, 1980).

Therefore, it can be deduced that statistics play an important role in all aspects of a trial, from planning, design, conduct, analysis through to reporting and publication. There is a plethora of literature discussing statistical methodologies in trials from a mathematical perspective. Less has been written about the trial team and other resources and how these may be influencing the statistical planning, design, conduct, analysis and reporting of trial data. Therefore, there remains a gap in the literature that needs to be addressed; systematically reviewing articles to fully comprehend, collate, and thematically synthesise the factors influencing the statistical aspects of trials is required.

This protocol will describe the methodological details for:

- conducting a comprehensive search for relevant articles,
- applying justified inclusion and exclusion (eligibility) criteria,
- evaluating and appraising the included studies,
- thematically synthesising the findings of the included studies.

### Research question

What are the factors influencing the statistical planning, design, conduct, analysis and reporting in trials?

### Rationale and aim of this review

The aim of this systematic review is to identify and describe factors contributing to and influencing the planning, design, conduct, analysis and reporting of trials, from a statistical perspective.

### Objectives

- To construct a systematic search strategy incorporating the relevant elements at the centre of the research question,
- To identify, evaluate and critically appraise peer-reviewed literature (and relevant non-peer reviewed sources) that discuss factors contributing to the statistical aspects of a trial,
- To develop an explanatory framework through a narrative thematic synthesis, for how these factors may influence the statistical planning, design, conduct, analysis and reporting of trials,
- To present recommendations (based on the findings of this review) for how the statistical aspects of trials can be improved.

## Methods

The protocol for this systematic review is not deemed eligible for registration in the PROSPERO database, as the outcome of this review is not a health-related one. This systematic review will investigate aspects of a trial methodological nature and will not investigate specific outcomes of a clinical relevance.

This protocol follows the PRISMA-P (Preferred Reporting Items for Systematic Reviews and Meta-Analysis Protocols; Moher *et al.*, 2015) guidelines. The PRISMA-P checklist can be found in Figshare ( Zaki *et al.*, 2020c).

### Criteria for considering studies for this review

***Phenomenon of interest***. The phenomenon of interest are the factors influencing the statistical planning, design, conduct, analysis or reporting of trials. 

Papers will be included if they discuss any one or more of the following factors:

- Roles, responsibilities, and tasks of key team members responsible for the statistical elements of the planning, design, conduct, analysis and reporting of trials
- Qualifications, training, knowledge, experience and professional development of key team members responsible for the statistical planning, design, conduct, analysis and reporting of trials
- Processes of communication and collaboration between key team members, and with others, responsible for the statistical planning, design, conduct, analysis and reporting of trials

Sources that do not meet the eligibility criteria will be excluded. If the key term 'trial' is not included in the full text, the article is not included in the final stage of the screening process. 

While novel statistical methodologies, for example novel trial designs, can contribute to the statistical aspects of a trial, the focus of this review is on other factors influencing the planning, design, conduct, analysis and reporting of a trial.

Articles will be excluded if they focus solely on:

- Pre-clinical research studies (animal/non-human studies)
- Evaluation of effectiveness of an intervention for the prevention or treatment or clinical diseases/conditions
- Cost-effectiveness articles or ones of a health economics perspective, not influencing the statistics of a trial
- Regulatory articles, with no reference to factors influencing the statistical planning, design, conduct, analysis or reporting of randomised trials
- Statistical theory

***Types of studies***. We will include all primary studies of any study design including studies that collect or analyse primary data through means of a secondary analysis. A preliminary scope of the literature revealed a lack of empirical literature in this field and therefore we will also include discussion/commentary papers.

### Search methodology

Keyword search terms are informed by conducting a preliminary scope of the literature. The search strategy for this review is created and refined with the assistance of an information specialist (health sciences liaison librarian), experienced in systematic reviews. Key words are first identified, followed using controlled vocabulary search terms in individual databases.

The SPICE (Setting – Perspectives – Intervention – Comparison – Evaluation) framework is considered to be the best fit for the search strategy required in this systematic review, as the individual search features are suitable for the type of search in this review (see below). Other frameworks such as PICO (Population-Intervention-Comparison-Outcome), SPIDER (Sample-Phenomenon of Interest-Design-Evaluation-Research Type) and CIMO (Context-Intervention-Mechanism-Outcome) ( Booth, 2016) were considered but not deemed appropriate. The search strings (for S, P, I and E) can be found in Table 1 below. An optional search string, Comparison (C), is not included, as the aim is not to compare interventions or study designs but to extract information from each article.

**Table 1.** Keyword search terms.

Feature	Search Terms
**Setting (S)**	"clinical research"
**Perspective (P)**	Statistician* OR biostatistician* OR "data manager*" OR investigator* OR "trial team*" OR "clinical research team*" OR "statistics team*" OR "data management team*"
**Intervention (I)**	"clinical trial*" OR trial OR trials
**Evaluation (E)**	Statistics OR biostatistics OR data OR statistical OR planning OR design OR conduct OR analysis OR analyses OR reporting

While the Setting (S) of the search terms is "clinical research", the Intervention (I) and focus of the review is on trials, not other clinical studies such as observational research. With regards to the Evaluation (E), the key elements from the research question (planning, design, conduct, analysis (or the plural ‘analyses’) and reporting) are included as these are the aspects to be evaluated and from where the relevant factors can be extracted. As statistics is the central phenomenon of this systematic review, the terms ‘statistics’ and ‘statistical’ are crucial. An alternative term for statistics, ‘biostatistics’ is included to ensure articles that mention this term instead of ‘statistics’ are not missed. Similarly, the search term ‘data’ is included to retrieve articles discussing data aspects that may influence the statistics of a trial.

Within each of the search term features, the keywords are combined using the ‘OR’ Boolean operators between them. This allows for broadening the search. Methods used to narrow the search while also using the Boolean operator includes the concept ‘AND’ between the features (see Table 1). Before the search is run in its entirety, search terms are individually assessed and run through the databases.

A systematic literature search is performed on the following electronic databases: PubMed NCBI, Web of Science, PsycINFO and CINAHL. This search in the bibliographic databases retrieves peer reviewed literature.

A pre-defined set of controlled vocabulary terms are used in the individual databases. These terms index, describe and categorise sources of information and ensures a comprehensive search in the databases. In the CINAHL database, such controlled vocabulary terms are known as 'Subject Headings'. In PubMed, the Advanced Search Builder finds alternative terms used by that database to describe a term. Such terms are known as MeSH (Medical Subject Headings). The list of controlled vocabulary terms in the PyscINFO database are known as the 'Thesaurus of Psychological Index Terms'. Web of Science is the only database included in this search strategy that does not have controlled vocabulary terms. If controlled vocabulary terms are appropriate and relevant, they are included for that database. Articles of interest retrieved in the preliminary scope of the literature are also used to locate MeSH terms of relevance in PubMed. These controlled vocabulary terms, while often the same as the original keyword search terms, are included to maximise the retrieval of potentially relevant articles. Individual MeSH/controlled vocabulary/thesauri terms from the individual databases can be found in *Extended Data* ( Zaki *et al.*, 2020a).

To ensure a comprehensive search is conducted, the review is not limited geographically and so there are no restrictions on the locations/countries where articles are published.

The searches are also not limited by publication date. No database filters or restrictions are placed on gender or age of participants discussed in the articles. Due to limited translation resources, searches are limited to only include articles written in English. This systematic review is limited to peer-reviewed articles retrieved in the bibliographic databases and non-peer reviewed sources from the grey literature.

### Information sources

While it is expected that most of the articles relevant for this systematic review can be obtained from the systematic bibliographic database search discussed, important sources may also be retrieved from grey searches. This search will therefore retrieve unpublished or non-peer reviewed literature sources. Open Grey EU is the grey literature database that will be used in this review. Backwards citation screening will also be conducted in this review. Relevant articles from the reference list of included articles, that meet the eligibility criteria, are included.

Guidelines relevant to the scope of the review are also important sources of information. Guidelines that will be included in this systematic review are:

- Food and Drug Administration (FDA)’s 1988: Format and content of the clinical and statistical sections of an application
- Statisticians in the Pharmaceutical Industry (PSI) 1994: Guideline for standard operating procedures (SOPs) for good statistical practice in clinical research
- International Conference on Harmonisation Good Clinical Practice (ICH GCP) 1998: Topic E 9 statistical principles for clinical trials
- American Statistical Association (ASA) 2018: Ethical guidelines for statistical practice
- Consolidated Standards of Reporting Trials (CONSORT) 2010: Checklist for reporting randomised controlled trials (RCTs)

The FDA, PSI, ICH GCP, ASA and CONSORT websites will be listed as professional organisations.

If additional information (not explicitly stated in the articles) is required, direct contact will be made with the study authors of the articles.

### Data collection and analysis

***Selection of studies***. Two reviewers (MZ and LOS) will independently screen title and abstracts against the inclusion criteria. Disagreements will be resolved through discussion with a third reviewer (EM). The full text of papers included at title and abstract stage will also be assessed by two reviewers with recourse to a third (EM) should disagreements occur. A summarised synthesis will be provided of reasons all papers at full text stage were excluded.

***Data extraction and management***. Citations from database searches will be imported into EndNote and then merged and de-duplicated. Microsoft Excel will be used for data extraction. 

Key characteristics and important features of the included articles will be extracted using a previously piloted structured data extraction form, in Microsoft Excel (template provided in *Extended Data* ( Zaki *et al.*, 2020b)). In brief, this file presents the key features (‘data items’, in accordance with PRISMA-P) that will be extracted from the full text of the included articles. 10–20% of the data extraction will be checked for accuracy and thoroughness by a second researcher (EM).

### Quality assessment

Once the researcher is aware of the types of articles to be included in the systematic review, it will then be decided whether the Critical Appraisal Skills Programme ( CASP, 2020) checklists or Mixed Methods Appraisal Tools ( MMAT, 2018) will be used to assess the quality of included articles. This will determine the strength of the body of evidence. The results of this appraisal will then be used to inform the synthesis and the interpretation of the results as well as the discussion of the findings of this review.

### Data analysis and synthesis

As this review is not aiming to assess the risk of bias in any particular intervention or appraise the methodological quality of trials, the data analysis and synthesis will be limited to a thematic/narrative synthesis approach. While quantitative articles will be included in the review, their quantitative outcomes will not be assessed for heterogeneity or quantitatively synthesised in the form of a meta-analysis. There is also no planned assessment for meta-biases in the studies that are included in this review.

The aim of the data extraction, analysis and synthesis process is to extract factors influencing statistics, statistical processes, statistical interactions, and other statistical aspects in all stages of a trial. For this review, ‘factors’ can be defined as any element impacting, influencing, or contributing to any aspect of statistical planning, design, conduct, analysis and reporting in trials. A narrative thematic synthesis approach will be used to collate and describe the key information from all included sources. This approach has been used to analyse data and information extracted from both quantitative and qualitative studies ( Ryan *et al.*, 2018).

Each stage of the analysis will be documented, as it is important to maintain transparency about how patterns within the data are identified, and to note any similarities or differences between sources. A coding frame will be presented. Similarities between codes will be identified, labelled and categorised into themes. Narrative descriptions of each theme will be provided. The final outcome of the thematic synthesis will be an overall account of the factors influencing the statistical planning, design, conduct, analysis and reporting in trials. Findings across articles will be synthesised and their meanings interpreted to answer the research question ( Ryan *et al.*, 2018). Recommendations, where provided in the articles, will be emphasised. This review will also illuminate areas that are under-researched and where there is scope for future work. One reviewer (MZ) will undertake each of the steps of data extraction, synthesis and comparison of findings across information sources, and the work will be checked by a second researcher (EM). Feedback from a Research Studies Panel (MG, DD, EM) will be included in the final version of the systematic review.

### Potential limitations

This review will only include studies that are published in English, due to limitations in translation resources. This could mean excluding other relevant information based on language barriers. Secondly, unpublished literature will not be included, possibly leaning towards an increased risk of publication bias in the research that is included.

### Tracked and dated amendments

Any amendments to this protocol, including the dates of the amendments and justifications, will be documented and presented in a table in the systematic review publication.

## Dissemination

The findings of the systematic review will be published in a peer-reviewed journal upon completion. This systematic review will be of interest not only to statisticians (biostatisticians) and data managers, but also PIs and other researchers and healthcare professionals working in the trials field.

## Conclusion

The aim of this systematic review is to identify, describe and synthesise factors contributing to or influencing the statistical planning, design, conduct, analysis and reporting of trials. This will be conducted through a systematic search of the literature in four bibliographic databases: PubMed NCBI, Web of Science, PsycINFO and CINAHL. The eligibility criteria for including relevant articles is discussed along with the plans for critical appraisal, data extraction and data synthesis. The systematic review and thematic synthesis will be written in accordance with the PRISMA statement for systematic reviews. The data extraction and findings will be tabulated to present key features. Findings will then be synthesised narratively. The findings will inform healthcare professionals and researchers of key factors revolving around trial statistics and whether such factors can be addressed to better understand the methodology of trials.

## Data availability

### Underlying data

No data is associated with this article.

### Extended data

Figshare Repository: Controlled Vocabulary Search Terms Bibliographic Databases, https://doi.org/10.6084/m9.figshare.12403421.v1, ( Zaki *et al.*, 2020a).

Figshare Repository: Data Extraction Template for Systematic Review, https://doi.org/10.6084/m9.figshare.12403562.v1, ( Zaki *et al.*, 2020b).

### Reporting guidelines

Figshare: PRISMA-P checklist for “A protocol for a systematic review investigating factors influencing statistical planning, design, conduct, analysis and reporting of clinical trials”, https://doi.org/10.6084/m9.figshare.12264938.v1 ( Zaki *et al.*, 2020c).

Data are available under the terms of the Creative Commons Zero “No rights reserved” data waiver (CC0 1.0 Public domain dedication).
