# Supplementary material for: A protocol for a systematic review investigating the factors influencing the statistical planning, design, conduct, analysis and reporting of trials
Source: HRB Open Res. 2020 Sep 24;3:36. Originally published 2020 Jun 8. [Version 2] doi: 10.12688/hrbopenres.13068.2 (PMC7445680; doi:10.12688/hrbopenres.13068.2)
Supplement: Peer review history

# Peer review: Reviewer response for version 1

**DOI:** 10.21956/hrbopenres.14167.r27784

**Published:** 2020-08-24

**Authors:** Basil Elnazir, Nadeem Gadelseyed

**Affiliations:** 1 Trinity College Dublin, Dublin, Ireland; 2 Tallaght University Hospital, Dublin, Ireland

I have asked Dr Nadeem Gadeleseyd to independently review this article with me and we both reached the below conclusion.

 This study sets out to identify, describe and synthesise factors contributing to or influencing the statistical planning, design, conduct, analysis and reporting of clinical trials.

 The article is clearly written and easy to follow. The search methodology is clear and using standard tools.

 Below are few pointers the authors should attend to:

1. The inclusion criteria are a bit vague and confusing and I suggest rewriting this section with clear determinants and tight criteria to avoid selection bias.
2. The authors should describe the reason for selecting this timeline. 'The timeline for gathering sources to be included in this systematic review is from April to June 2020'.
3. It will be helpful if the authors would eventually consider a plan to construct a protocol template to better inform researchers and healthcare professionals around statistical aspects, planning, design, conduct, analysis and reporting of clinical trials.

Is the study design appropriate for the research question?

Yes

Is the rationale for, and objectives of, the study clearly described?

Yes

Are sufficient details of the methods provided to allow replication by others?

Yes

Are the datasets clearly presented in a useable and accessible format?

Yes

Reviewer Expertise:

Childhood Asthma, Cystic fibrosis and sleep disordered breathing.

We confirm that we have read this submission and believe that we have an appropriate level of expertise to confirm that it is of an acceptable scientific standard.

# Response

**Published:** 2020-09-18

**Authors:** Marina Zaki

**Affiliations:** University College Dublin, Ireland

The authors would like to greatly thank Professor Elnazir and Dr. Gadelseyed for their constructive comments and suggestions on this protocol. We acknowledge the reviewer's comments regarding the eligibility criteria and avoiding selection bias. The new version of the protocol has been amended to reflect this. As this systematic review was part of a wider doctoral study, the timeline for gathering sources was described. However this statement has been removed in the revised version.

# Peer review: Reviewer response for version 1

**DOI:** 10.21956/hrbopenres.14167.r27785

**Published:** 2020-08-10

**Authors:** Dawid Pieper

**Affiliations:** 1 Institute for Research in Operative Medicine, Faculty of Health, School of Medicine, Witten/Herdecke University, Cologne, Germany

This is a well written protocol. I have only a few comments.

 In your objectives you state that you are also interested in factors related to reporting of trials. What do you mean by that? Is that the reporting of the whole trials or does this only relate to statistical aspects. If the latter is the case, I doubt that this can be differentiated clearly as this would not only be about the methods section but also about the results and its interpretation.

 In your title you state that your study is about clinical trials. This is not stated clearly in your eligibility criteria where you refer to randomised trials which is not exactly the same. Are you intending to exclude trials in psychology? Psychology might be a completely different field as psychologists are well educated in methods and statistics (in contrast to clinicians). What I am trying to say is that an average psychologist will be able to run a better methodological trial on your own than an average clinician. The latter does need the help of a statistician.

 Did you involve an information specialist in drafting your search strategy? Was the search strategy checked using the PRESS guideline? I feel that searching for "clinical research" is too narrow. The term "clinical trial*" can be omitted as you are searching for "trial" and "trials" anyway. At other instances I would suggest to search for "conducting*" instead of "conduct" (the same does apply to other terms).

 All in all, identifying these articles might be challenging. What about forward and backward citation screening?

 I wonder about the statement that the literature will be gathered between April and June 2020 only. This would mean that the search did already take place. While I understand that researchers cannot wait to go on with their study waiting for the protocol to be published for several reasons, I would still advise the authors to check their search strategy and information sources.

Is the study design appropriate for the research question?

Yes

Is the rationale for, and objectives of, the study clearly described?

Yes

Are sufficient details of the methods provided to allow replication by others?

Yes

Are the datasets clearly presented in a useable and accessible format?

Not applicable

Reviewer Expertise:

Methodologist, clinical epidemiologist, meta-researcher.

I confirm that I have read this submission and believe that I have an appropriate level of expertise to confirm that it is of an acceptable scientific standard.

# Response

**Published:** 2020-09-18

**Authors:** Marina Zaki

**Affiliations:** University College Dublin, Ireland

The authors would like to greatly thank Dr. Pieper for the constructive comments on this protocol. The authors would like to clarify and address a number of points:

1) We agree that it is difficult to differentiate between the reporting of whole trials and statistical reporting (methods, results and interpretation). Therefore, we would like to clarify that we have included articles discussing the reporting of trials, in the context of statisticians/investigators roles and responsibilities, and therefore this relates back to the research question of factors influencing statistics.

2) The new version of the protocol has been revised, to address the comment regarding the eligibility criteria. The term 'trial' has now replaced 'randomised trial' and 'clinical trial', to capture the breadth of articles included in this review.

3) An information specialist was involved when drafting the search strategy. The authors acknowledge that the PRESS guideline and truncation could have been used. However, a search with the suggested truncated terms did not reveal additional articles.

4) The authors acknowledge the importance of backwards citation screening and so this will be conducted. The protocol has been amended to reflect this. 

5) As this systematic review is part of a doctoral study, the aim was to have sources gathered by the stated timeline. However, this statement has now been removed and the authors acknowledge the comments about checking the information sources and search strategy.
